# Integrated analysis of gut metabolome, microbiome, and exfoliome data in an equine model of intestinal injury

**DOI:** 10.1186/s40168-024-01785-1

**Published:** 2024-04-15

**Authors:** C. M. Whitfield-Cargile, H. C. Chung, M. C. Coleman, N. D. Cohen, A. M. Chamoun-Emanuelli, I. Ivanov, J. S. Goldsby, L. A. Davidson, I. Gaynanova, Y. Ni, R. S. Chapkin

**Affiliations:** 1 Department of Large Animal Clinical Sciences, College of Veterinary Medicine & Biomedical Sciences, Texas A&M University, College Station, TX USA; 2 Department of Statistics, College of Arts & Sciences, Texas A&M University, College Station, TX USA; 3 Department of Veterinary Physiology and Pharmacology, College of Veterinary Medicine & Biomedical Sciences, Texas A&M University, College Station, TX USA; 4 Program in Integrative Nutrition & Complex Diseases, College of Agriculture & Life Sciences, Texas A&M University, College Station, TX USA; 5 Mathematics & Statistics Department, College of Science, University of North Carolina Charlotte, Charlotte, NC USA

**Keywords:** Host-microbiota interactions, Exfoliome, Metabolome, Mucosal transcriptome, Oxidative stress, Non-invasive, Computational biology

## Abstract

**Background:**

The equine gastrointestinal (GI) microbiome has been described in the context of various diseases. The observed changes, however, have not been linked to host function and therefore it remains unclear how specific changes in the microbiome alter cellular and molecular pathways within the GI tract. Further, non-invasive techniques to examine the host gene expression profile of the GI mucosa have been described in horses but not evaluated in response to interventions. Therefore, the objectives of our study were to (1) profile gene expression and metabolomic changes in an equine model of non-steroidal anti-inflammatory drug (NSAID)-induced intestinal inflammation and (2) apply computational data integration methods to examine host-microbiota interactions.

**Methods:**

Twenty horses were randomly assigned to 1 of 2 groups (*n* = 10): control (placebo paste) or NSAID (phenylbutazone 4.4 mg/kg orally once daily for 9 days). Fecal samples were collected on days 0 and 10 and analyzed with respect to microbiota (16S rDNA gene sequencing), metabolomic (untargeted metabolites), and host exfoliated cell transcriptomic (exfoliome) changes. Data were analyzed and integrated using a variety of computational techniques, and underlying regulatory mechanisms were inferred from features that were commonly identified by all computational approaches.

**Results:**

Phenylbutazone induced alterations in the microbiota, metabolome, and host transcriptome. Data integration identified correlation of specific bacterial genera with expression of several genes and metabolites that were linked to oxidative stress. Concomitant microbiota and metabolite changes resulted in the initiation of endoplasmic reticulum stress and unfolded protein response within the intestinal mucosa.

**Conclusions:**

Results of integrative analysis identified an important role for oxidative stress, and subsequent cell signaling responses, in a large animal model of GI inflammation. The computational approaches for combining non-invasive platforms for unbiased assessment of host GI responses (e.g., exfoliomics) with metabolomic and microbiota changes have broad application for the field of gastroenterology.

Video Abstract

**Supplementary Information:**

The online version contains supplementary material available at 10.1186/s40168-024-01785-1.

## Background

The mammalian gastrointestinal (GI) tract is a complex system both anatomically and physiologically that is further complicated by the vast collection of microorganisms inhabiting it. It is well appreciated that combinatory biology of the host (GI) tract and microbiota play an essential role in the digestion of nutrients and production of energy [1, 2]. Importantly, changes in the microbiota have been linked to a diverse array of both GI and non-GI health conditions in people and animals and there are several reviews that describe these associations [3, 4]. In human and veterinary medicine, the decreasing cost and increasing availability of culture-independent approaches (i.e., next generation sequencing) to study the microbiome have resulted in a wealth of descriptive studies examining the microbiota in the context of health and disease. These studies augment our understanding of how the composition of the microbiota can be altered in various disease states. Studies linking these microbial changes to host function are uncommon, however, because they are challenging to conduct. Without the combination of both microbial and host data, it remains unclear whether microbiomic changes are the cause or effect of a disease, limiting the utility of the information. Thus, the ability to sequentially interrogate changes in both the host and microbiome is needed to unravel the complex interplay between the host and the microbiome.

While non-invasive coprological approaches have been widely used to capture information regarding the microbial niche, there is a paucity of non-invasive approaches to capture similar information regarding host function. One such approach is the use of exfoliomics. This platform has been utilized in rodents [5], pigs [6], adult humans [7], and human neonates [8]. We recently also validated this approach in horses [9]. Non-steroidal anti-inflammatory drug (NSAID)-induced intestinal injury (i.e., enteropathy) is a clinical syndrome widely recognized in human medicine with a similar disease in animals albeit different anatomic sites affected depending on the animal species [5, 10, 11]. NSAID-induced intestinal injury of mice, rats, and pigs has been used as a model system for studying inflammatory bowel disease (IBD) in people [12–14]. Both the clinical syndrome and the model are characterized by microbiota changes, neutrophilic intestinal inflammation, and gross intestinal lesions ranging from subclinical evidence of mucosal injury to potentially fatal intestinal bleeding and perforations [15, 16]. We have developed an equine model of NSAID-induced intestinal inflammation [17–19], which mirrors the microbiota changes and damage to both the upper and lower GI tract observed with NSAID-induced intestinal injury in both clinical cases and other animal models. Moreover, the inducible, mild, predictable, and reversible nature of this model make it an attractive platform to examine the intersection of host and microbial function in the context of GI intestinal injury. Importantly, the severity of injury is mild. Therefore, changes in the microbiome and host gene expression are not masked by the overwhelming inflammatory cascade that accompanies more severe injury. In addition, use of this model has potential clinical benefits for horses as any information gained about host and microbiota interactions could be leveraged to develop preventative or treatment strategies for GI diseases of horses. This is important because GI diseases, including colic and colitis, are of considerable importance to horses and the horse industry, second only to old age as a cause of death [20]. Further, gaining information about the equine GI tract is challenging from a clinical perspective due to the immense size of the horse, which precludes the use of advanced imaging modalities and the acquisition of diagnostic endoscopic biopsies in many cases. Thus, use of the equine model of NSAID-induced intestinal inflammation not only enables a novel platform for understanding host-microbiota interactions in the context of GI disease across species but also can aid in the identification of mechanisms for preventing GI disease and NSAID-induced injury in horses.

Another major limitation that has hampered understanding of host-microbiota interactions is the challenging computational analysis of large omic datasets. High-dimensional data are inherently noisy, and this becomes even more problematic when the sample size is small [21]. Here, we attempted to overcome this challenge by application of multiple computational approaches to select features that were commonly identified by all approaches. Taken together, our model and computational analysis provides a potential platform for elucidating host-microbiota interactions by identifying initiating events of injury in a robust and accurate manner. Our objectives were to first characterize changes in the host gene expression profiles, fecal microbiota, and fecal metabolome changes in an equine model of intestinal inflammation and then to apply multiple methods of computational data integration to examine host-microbiota interactions in the context of GI inflammation.

## Methods

### Study design

The protocol for this study was approved by the university Institutional Animal Care and Use Committee (IACUC 2018–003). The equine model of NSAID-induced intestinal injury was performed, as previously described [17–19]. Briefly, twenty healthy adult horses from the university herd were utilized for this study. Pairs of horses were matched based on breed, age (± 2 years), weight (± 45 kg), and sex. One horse from each pair was randomly assigned to either the control group or the NSAID group. The two groups were housed in separate but neighboring pastures. There was a 75-day acclimation period prior to the 9-day model (Supp. Figure 1). During both the acclimation period and the treatment period, all horses were managed identically. Horses were confined to sand dry lots only and thus no grazing occurred. The diet consisted of free-choice coastal Bermuda hay all from the same cutting and free choice water. On day 0, baseline feces and blood was collected and gastroscopy was performed (see below). Beginning on day 1, the NSAID phenylbutazone^a^ was administered [4.4 mg/kg orally q24 hours] to the NSAID group and horses assigned to the control group were given an equivalent volume of placebo (base of phenylbutazone paste). These treatments were administered for 9 days. On day 10, fecal and blood samples were collected and gastroscopy repeated. A physical examination was performed on all horses each day during the 9 days of phenylbutazone administration. Rectal temperature, heart rate, and respiratory rate were recorded. The dosage of phenylbutazone was chosen based on label directions as this dosage is frequently used to manage common inflammatory conditions in horses (e.g., osteoarthritis) [22–24].

### Gastroscopy, fecal collection, and blood collection

Fecal samples were collected by rectal palpation using one rectal sleeve per animal on days 0 and 10. Feces were collected in a sterile container, immediately placed on dry ice, and transferred to a − 80 °C freezer for long-term storage. For exfoliomic analysis, an additional 1 g of feces was homogenized in 20 mL of RNA Shield® (Zymo Research, Irvine, CA, USA) and stored at − 80 °C until processed (see below). Whole blood (10 mL) was collected on days 0 and 10 from an aseptically prepared jugular vein. Blood was collected in a serum separator tube (Becton, Dickinson and Company, Franklin Lakes, NJ, USA) and processed within 60 min. Serum was collected after centrifugation (1000 RCF, 10 min, 20 °C) and stored at − 80 °C until utilized for an ELISA (see below).

Gastroscopy was performed on days 0 and 10 as previously described [18]. Briefly, each horse was held off feed for 18 h and water for 3 h before gastroscopy. Horses were sedated using xylazine hydrochloride (0.4 mg/kg IV) and a 3-m endoscope was passed into the stomach. The entire stomach was examined, including the pylorus, and assigned a score by a single observer board certified in large animal internal medicine and blinded to treatment group. Squamous scoring was based on a previously published scoring system: 0 = intact normal mucosa; 1 = intact mucosa with reddening, hyperkeratosis, or both; 2 = small single or small multifocal ulcers; 3 = large single or large multifocal ulcers; and 4 = extensive (often coalescing) ulcers with areas of deep ulceration [25]. Glandular ulcers were scored using the same criteria as described for squamous ulcers (without consideration of lesion depth).

### Tumor necrosis factor ELISA

Tumor necrosis factor (TNF) was quantified from serum on days 0 and 10 using a commercially available kit (R&D Systems, Minneapolis, MN, USA), according to manufacturer’s protocol.

### Metabolome

Global non-targeted mass spectrometry metabolomics analysis was performed at Metabolon, Inc (Metabolon, Inc, Durham, NC), a commercial supplier of metabolic analysis, which has developed a platform that integrates chemical analysis (including identification and relative quantification) and quality assurance. To maximize compound detection and accuracy, 3 separate analytical methods were utilized including ultra-high performance liquid chromatography-tandem mass spectrometry (UHPLC-LC–MS) in both positive and negative ion modes and gas chromatography/mass spectrometry (GC–MS) [26, 27]. Targeted analysis and quantification of eight short chain fatty acids (SCFA) was determined with LC–MS/MS.

Sample preparation was performed by the automated Mircolab STAR system (Hamilton Company, Salt Lake City, UT, USA). To remove, dissociate small molecules, and to recover chemically diverse metabolites, proteins were precipitated with methanol under vigorous shaking for 2 min (GenoGrinder 2000, Glen Mills, Clifton, NJ, USA) followed by centrifugation. The resulting extract was placed briefly on a TurboVap® (Zymark Corporation, Hopkinton, MA, USA) to remove the organic solvent. The sample extracts were stored overnight under nitrogen before preparation for analysis. Bioinformatics for metabolite data consisted of 4 components, the Laboratory Information Management System (LIMS), the data extraction and peak-identification software, data processing tools for QC and compound identification, and a collection of information interpretation and visualization. These analyses were all performed on the LAN backbone, and a database server running Oracle 10.2.0.1 Enterprise Edition. Prior to analysis, values were normalized in terms of raw area counts and the rescaled to set the median equal to 1.

### Microbiota

DNA extraction, 16S rRNA gene PCR, and sequencing were performed, as previously described in a separate publication [18]. Briefly, 200 mg of feces was chipped from the frozen fecal sample and genomic DNA was isolated using a commercially available fecal DNA isolation kit (QIAamp® Fast DNA Stool Mini Kit, Qiagen, Germantown, MD, USA) according to manufacturer’s protocol with slight modification. The modifications included a bead beating step with 50 mg each of sterile DNAase-free 0.1- and 0.5-mm silica zirconium beads for 90 s at 6 m/s using a Bead Mill Homogenizer (VWR, Radnor, PA, USA). The sample then was heated at 70 °C for 10 min. The remainder of the protocol was performed according to manufacturer’s protocol.

Amplification and sequencing of the V3-V4 variable region of the16S rRNA gene was performed commercially (Zymo Research, Irvine, CA, USA). Briefly, a library was prepared using a commercially available 16S rRNA prep kit (Quick-16S NGS Prep Kit, Zymo Research, Irvine, CA, USA), samples were barcoded, and PCR primers for the V3-V4 hypervariable region of the 16S rRNA gene were used. Sequencing was performed on a MiSeq (Illumina, San Diego, CA, USA) following the manufacturer’s guidelines. The software Quantitative Insights Into Microbial Ecology (QIIME2—ver 2019.1) (https://qiime2.org), dada2 (ver 1.6), and phyloseq (ver 1.28.0) were used for data processing and analysis [28–30]. Sequences were quality filtered and assigned to amplicon sequence variant (ASV) using dada2. Qiime2 was used to assign taxonomy to these ASVs against the Greengenes database (ver. gg_13_8) filtered at 97% identity for 16S rRNA gene sequences. Count tables with assigned taxonomy and phylogenetic trees constructed in QIIME2 were exported to R (ver. 3.6.1). Phyloseq was used to collapse ASV tables to the genera level. Any genera that were present in 5 or fewer samples were removed. ASV genus level count tables were then exported for further analysis.

### Exfoliome

The global gastrointestinal transcriptome was assessed using exfoliomics from day 10 samples only. PolyA + RNA was isolated from fecal samples, as previously described [9]. Briefly, RNA was extracted using a commercially available kit (Active Motif, Carlsbad, CA, USA), quantified (Nanodrop spectrophotometer; Thermo Fisher Scientific, Waltham, MA, USA), and quality assessed (Bioanalyzer 2100; Agilent Technologies, Santa Clara, CA, USA). Each sample was processed with the NuGen Ovation 3′-DGE kit (San Carlos, CA, USA) to convert RNA into cDNA. Following cDNA fragment repair and purification, Illumina adaptors were ligated onto fragment ends and amplified to create the final library. Libraries were quantified using the NEBNext Library Quant kit for Illumina (NEB, Ipswich, MA, USA) and run on an Agilent DNA High Sensitivity Chip to confirm sizing and the exclusion of adapter dimers. Sequencing data were demultiplexed and assessed for quality using FastQC. Reads were aligned using Spliced Transcripts Alignment to a reference software with default parameters and referenced against the genome of the horse (EquCab 3.0) [31]. The resulting count table was used for subsequent statistical analysis.

### Data analysis

Several statistical models were used to identify variables to discriminate the control and NSAID groups in each dataset. Initially, the discriminatory power of each variable was evaluated with Model-Free Feature Screening for Ultrahigh Dimensional Discriminant Analysis (MV-SIS) [32]. MV-SIS measures individual variables’ ability to discriminate between the control and NSAID groups and produces a measurement that represents the discriminatory power of each variable. Subsequently, Multi-Group Sparse Discriminant Analysis (MGSDA) was performed [33]. This procedure jointly identifies discriminatory variables and estimates a subspace that separates the two groups based on identified variables. Unlike the marginal selection of MV-SIS, MGSDA accounts for the correlation structure of variables and selects only a subset of variables when informative variables are highly correlated. Lastly, we utilized Joint Association and Classification Analysis of multi-view data (JACA) to integrate data and classify [34]. JACA simultaneously identifies discriminative variables from the three data sets (microbiome, exfoliome, and metabolome). The selected variables from each data set provide coherent information to the model in that the signals corresponding to selected variables have high correlation across data sets.

To avoid data overfitting resulting in biased variable selection, we identified a set of informative variables based on out-of-sample prediction accuracy using Leave-One-Out Cross-Validation (LOO-CV). Specifically, for MGSDA and JACA, we fitted a model leaving one observation out and predicted the class (control or NSAID) of the left-out observation using the fitted model. We repeated this process for each observation and selected the set of variables that produced the smallest total number of misclassifications. The procedure was not applied to MV-SIS as it does not perform the variable selection, but rather provides a ranking for all variables in terms of their individual discriminatory power.

Graphical data presentation included principal component analysis (PCA) plots and Venn diagrams made in R (ver 4.1.3) with the R packages FactoMineR and FactoExtra and Venn. Random forest analysis was performed with the R package RandomForest. Gene pathway enrichment was determined using QIAGEN IPA (QIAGEN Inc., https://digitalinsights.qiagen.com/IPA) [35] by uploading appropriate gene lists with fold changes.

### Cell culture

For all in vitro assays, chemicals were obtained from Thermo Fischer Scientific (Waltham, MA, USA) unless otherwise noted. YAMC cells were kindly provided by Dr. Robert Whitehead [36]. Unless otherwise stated, YAMC cells were cultured in RPMI 1640 media containing GlutaMAX, Hepes and supplemented with 5% fetal bovine serum, ITS (Corning, Tewksbury, MA, USA), and mouse interferon gamma (Sigma-Aldrich, St. Louis, MO, USA).

### p62 nuclear translocation studies

To determine the effect of NSAIDs on nuclear translocation of p62, YAMC cells were seeded in 6-well plates (7.5 × 105 cells/well) and incubated under non-permissive conditions at 37 °C/5% CO_2_. The next day, media was replaced with media containing the appropriate treatments suspended in 0.04% dimethylformamide (VWR, Radnor, PA, USA), 0.4 mM ibuprofen (Cayman Chemical Co., Ann Arbor, MI, USA), 0.4 mM phenylbutazone (Cayman Chemical Co.), 0.25 mM indomethacin (Cayman Chemical Co.), 0.5 or 0.1 mM H_2_O_2_ and returned to the incubator. Twenty-four hours later, cells were washed once with Dulbecco’s phosphate-buffered saline (DPBS), detached, and transferred to a centrifuge tube. Cytosolic and nuclear fractions were collected, as previously described [37]. Briefly, samples were centrifuged at 500 × g for 10 min, washed once with 1 mL of DPBS and cell pellets were resuspended in 1440 µL of hypotonic solution (20 mM Tris–HCl (pH 7.4), 10 mM KCl, 2 mM MgCl_2_, 1 mM EGTA, 0.5 mM DTT, 0.5 mM PMSF). Samples were incubated on ice for 3 min, supplemented with NP-40 to a final concentration of 0.1%, and vortexed for 10 s vigorously prior to centrifugation at 3000 × g and 4 °C for 5 min. Supernatants containing cytosolic fractions were transferred into a clean tube and pellets were kept on ice for nuclear fraction isolation. Cytosolic containing supernatants were centrifuged at 15,000 × g and 4 °C for 3 min to remove any residual debris, transferred into a clean tube and stored at − 80 °C until western blot analysis. Pellets containing the nuclear fraction were washed once with isotonic solution supplemented with 0.3% NP-40 to remove any residual cytosolic proteins, centrifuged (3000 × g and 4 °C for 3 min) and lysed with 100 µL of radioimmunoprecipitation assay buffer (RIPA; 150 mM sodium chloride, 1.0% NP-40, 0.5% sodium deoxycholate, 0.1% sodium dodecyl sulfate, 50 mM Tris, pH 8.0) supplemented with protease inhibitor cocktail (Sigma-Aldrich, St. Louis, MO, USA). Samples were stored at − 80 °C until western blot analysis. The day of analysis, nuclear fractions stored in RIPA buffer were centrifuged at 3000 × g and 4° for 3 min before measuring protein concentration. Protein concentration was measured from the cytosolic and nuclear fractions using the bicinchoninic acid assay.

### Western blot analysis

Protein lysates (12.5–50 µg for cytosolic fractions and 10–15 µg for nuclear fractions) in 1X SDS/β-mercaptoethanol buffer were resolved on a 4–20% TGS stain free gel (BioRad, Hercules, CA, USA) and electrotransferred onto a polyvinylidene difluoride transfer membrane. Western blot analysis was performed using mouse anti-lamin A/C (1:2000; Cell Signaling Technology #4777, Danvers, MA, USA), rabbit anti-SQSTM1/p62 (1:1000; Cell Signaling Technology #5114) or rabbit anti-GAPDH (1:1000; Cell Signaling Technology #5174) and horseradish peroxidase-conjugated goat anti-rabbit (1:2000; Cell Signaling Technology #7074) or goat anti-mouse (1:5000, Abcam #ab6789, Waltham, MA, USA) antibodies. Protein bands were visualized by chemiluminescence using a ChemiDocTouch Imaging System (BioRad, Hercules, CA, USA). Bands were quantified using the ImageLab software version 5.2.1.

### Microscopy

YAMC cells were seeded in 2-well chamber cover glass (1.5 × 10^5^ cells/well) and incubated under non-permissive conditions (37 °C, 5% CO_2_). Thirty-six hours post seeding, media was replaced with the appropriate drug and cells were returned to the incubator. One-hour post drug addition, the reactive oxygen species (ROS) indicator (CM-H_2_DCFDA) was added to the wells at a final concentration of 2 ng/µL. Cells were returned to the incubator. One-hour post addition of ROS indicator, cells were washed once with media before imaging with a confocal laser scanning microscope (Olympus FV 3000, Shinjuku, Tokyo, JP). The ROS positive area was measured using Image J [38].

## Results

All horses completed the study but we were unable to collect one or both fecal samples from 2 horses (one from each group). Therefore, all fecal-based analyses are based on a sample size of 9 horses per group. All horses included in the study were geldings. The mean age in years ± SD for the control group and NSAID group was 14.7 ± 3.5 and 14.8 ± 3.2, respectively. Throughout the study, there was no clinical evidence of negative effects related to phenylbutazone administration, with vital parameters in all horses remaining within normal reference ranges. This is typical of the equine model of NSAID-induced intestinal injury [17]. All NSAID-treated horses in this study had prototypical evidence of subclinical intestinal injury including gastric ulcers and GI inflammation (Supp Figure 2).

### Individual data analysis

#### Metabolome

**Fig. 1:**
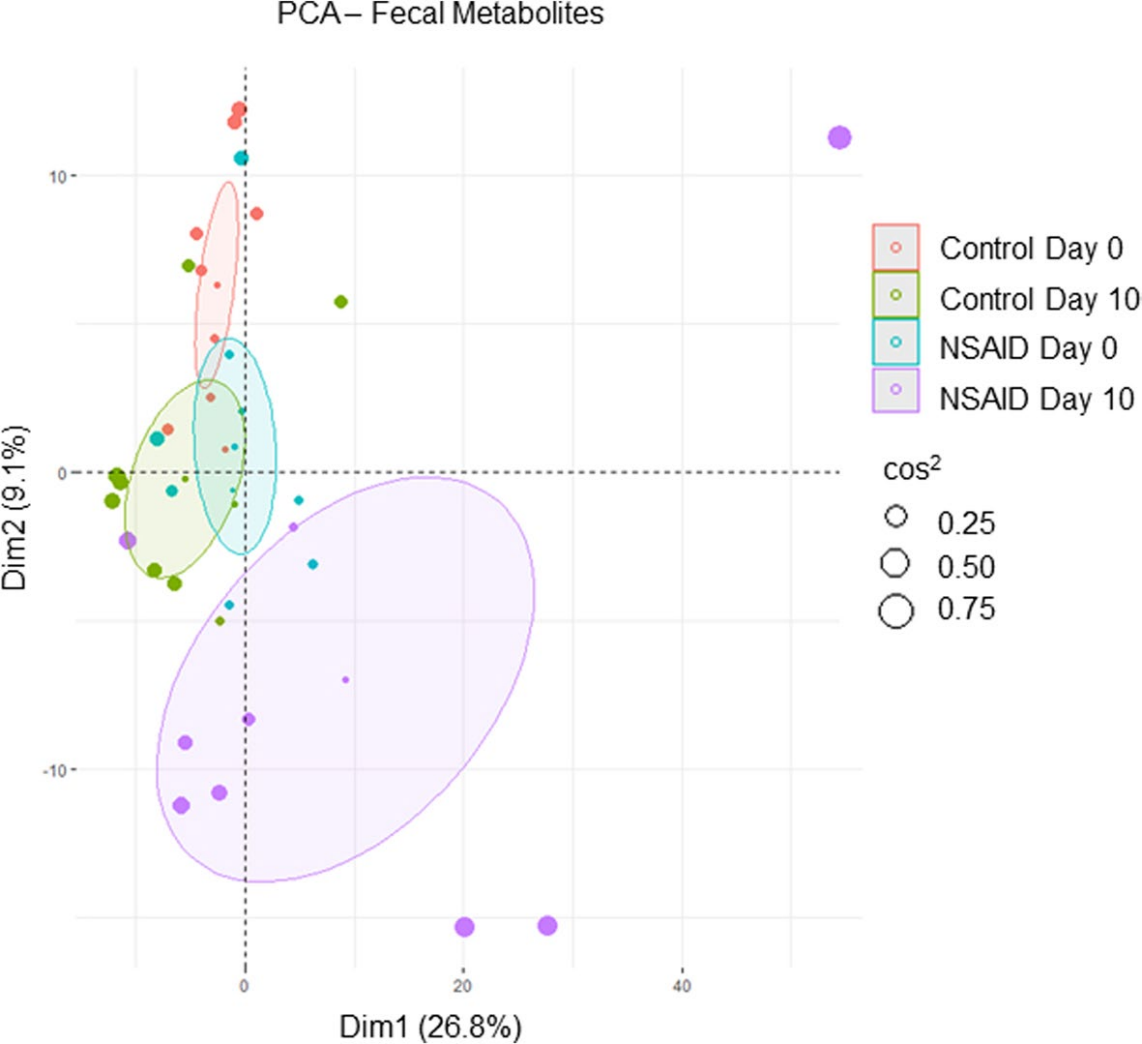
Fecal metabolome is altered by phenylbutazone administration. PCA of fecal metabolites grouped by treatment (NSAID or control) and day (day 0 = before NSAID administration and day 10 = after NSAID administration). Ellipses represent 95% CI around the group mean points. Point size indicates quality of representation (cos^2^) of individuals on the PCA; the larger point size reflects higher quality representation

Untargeted metabolomics was performed on fecal samples collected before and after 10 days of phenylbutazone administration for both the control and NSAID group. A total of 553 known compounds were identified (Supplemental Table 1). Phenylbutazone, only present in treated horses, was removed from the list of metabolites for all analyses. Principal component analysis (PCA) was performed to examine the ability of the entire fecal metabolome to separate the groups. There was overlap of all samples at day 0 but clear visual shifts of the fecal metabolic profile from day 0 to day 10, most notable in the NSAID group (Fig. 1). To further highlight differences in the fecal metabolome, random forest (RF) analysis was performed comparing control and NSAID groups at day 0 and day 10. RF analysis at day 0 resulted in an overall predictive accuracy of 60%, where a predictive accuracy of 50% would occur by chance alone. In contrast, following treatment, RF was 80% accurate at binning the samples.

**Fig. 2:**
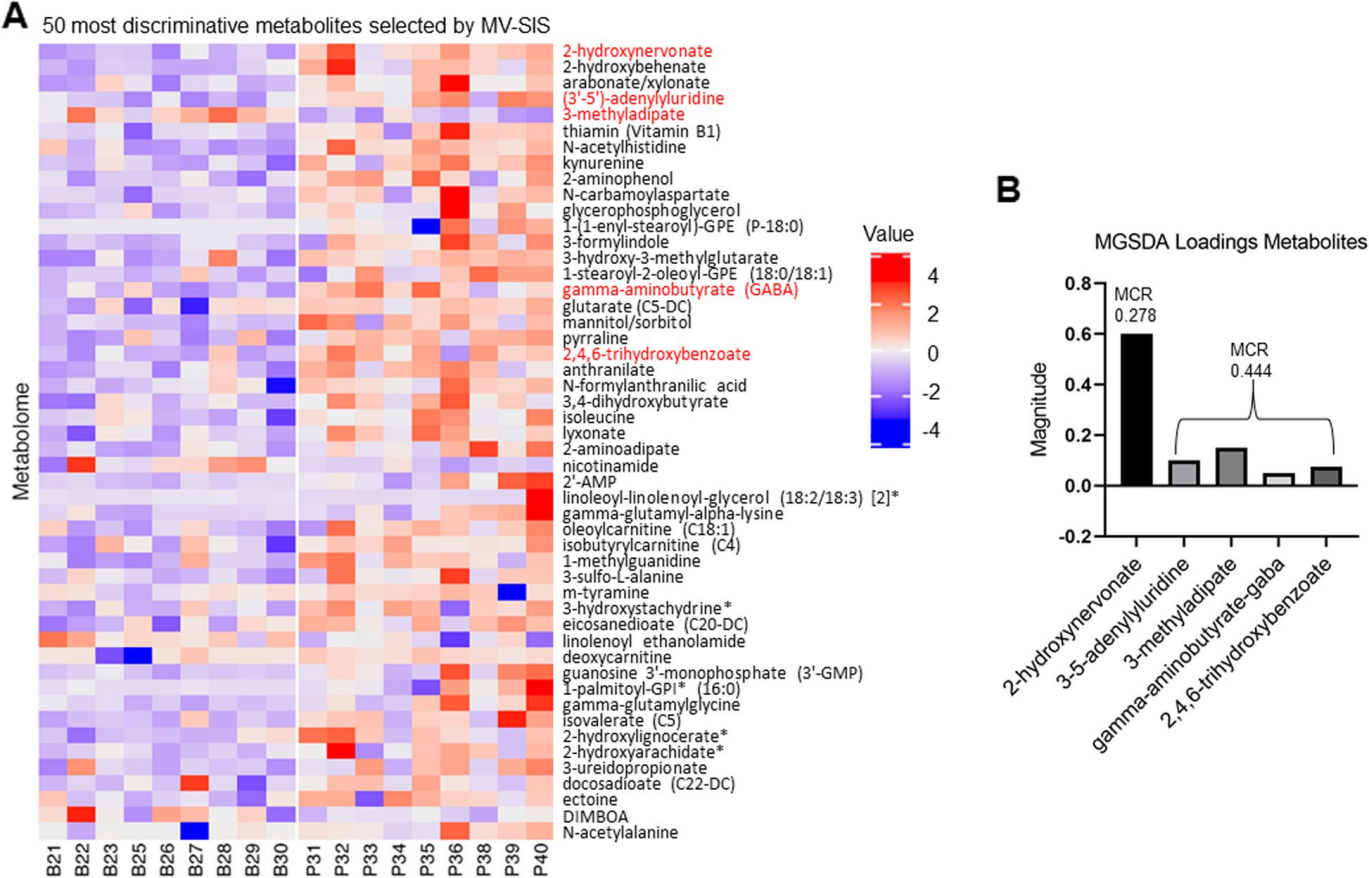
Specific fecal metabolites discriminate between control and NSAID-treated horses. **A** Heat map showing the distribution of the difference in fecal metabolites between day 10 and day 0 among the samples for the 50 most discriminative metabolites selected by MV-SIS. Values are scaled around zero as indicated by the key. Negative numbers (purple/blue) indicate lower concentration at day 10 compared to day 0 whereas positive numbers (orange/red) indicate higher concentration at day 10 compared to day 0. MGSDA-selected metabolites are shown in red text. **B** Bar chart indicating the magnitude of the loadings of MGSDA-selected metabolites. The misclassification rate (MCR) is indicated by the number at the top of each bar(s)

Next, feature selection was performed with MV-SIS, which screens for important predictors for the ultrahigh dimensional discriminant analysis with a categorical response. MV-SIS examines each variable (i.e., metabolite) individually and provides a number that represents the ability of that metabolite to discriminate between groups. These analyses were performed on data that represented the difference between day 10 and day 0 for each group. The 50 most informative metabolites selected by MV-SIS and their distribution among samples are shown in Fig. 2A. The top 300 features selected by MV-SIS were used for subsequent analyses (i.e., MGSDA and JACA). MGSDA jointly identifies discriminatory variables and, based on the variables, estimates a subspace that separates the two groups the most. Unlike the marginal selection of MV-SIS, MGSDA accounts for the correlation structure of variables and selects only a subset of variables when informative variables are highly correlated. Thus, if non-selected variables have high correlations (*R* > 0.9) with a MGSDA selected variable, then it is desired to investigate the non-selected variables because they have similar discriminatory power as the selected variable [33]. MGSDA identified the very long chain fatty acid (VLCFA) 2-hydroxynervonate as being the most informative metabolite. No additional metabolites were highly correlated with 2-hydroxynervonate. The average misclassification rate for this metabolite alone was 0.278. When MGSDA was allowed to select more variables, 4 additional metabolites were selected with an increased overall error rate (0.44) (Fig. 2B).

#### Microbiota

**Fig. 3:**
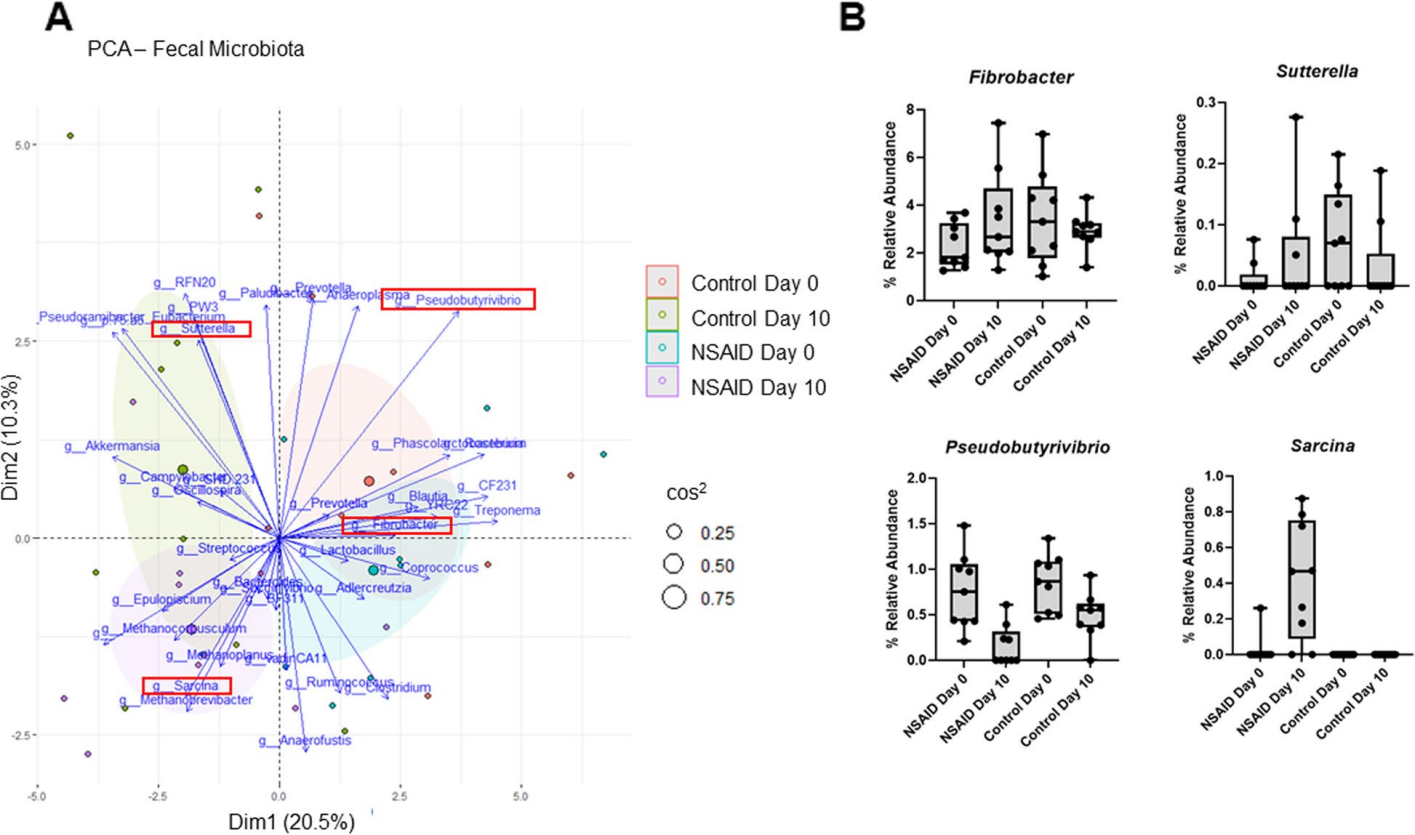
Fecal metabolome is altered by phenylbutazone administration. **A** PCA with biplot of fecal metabolites grouped by treatment (NSAID or control) and day (day 0 = before treatment and day 10 = after treatment). Ellipses represent 95% CI around the group mean points. Point size indicates quality of representation (cos^2^) of individuals on the PCA; the larger point size reflects higher quality representation. The 4 genera highlighted by the red box are the genera selected by subsequent analyses. **B** Boxplots showing the percent relative abundance of the 4 genera selected by subsequent analyses. Horizontal line represents the median, box extends from 25 to 75th percentiles, and whiskers extend to minimum and maximum values

**Fig. 4:**
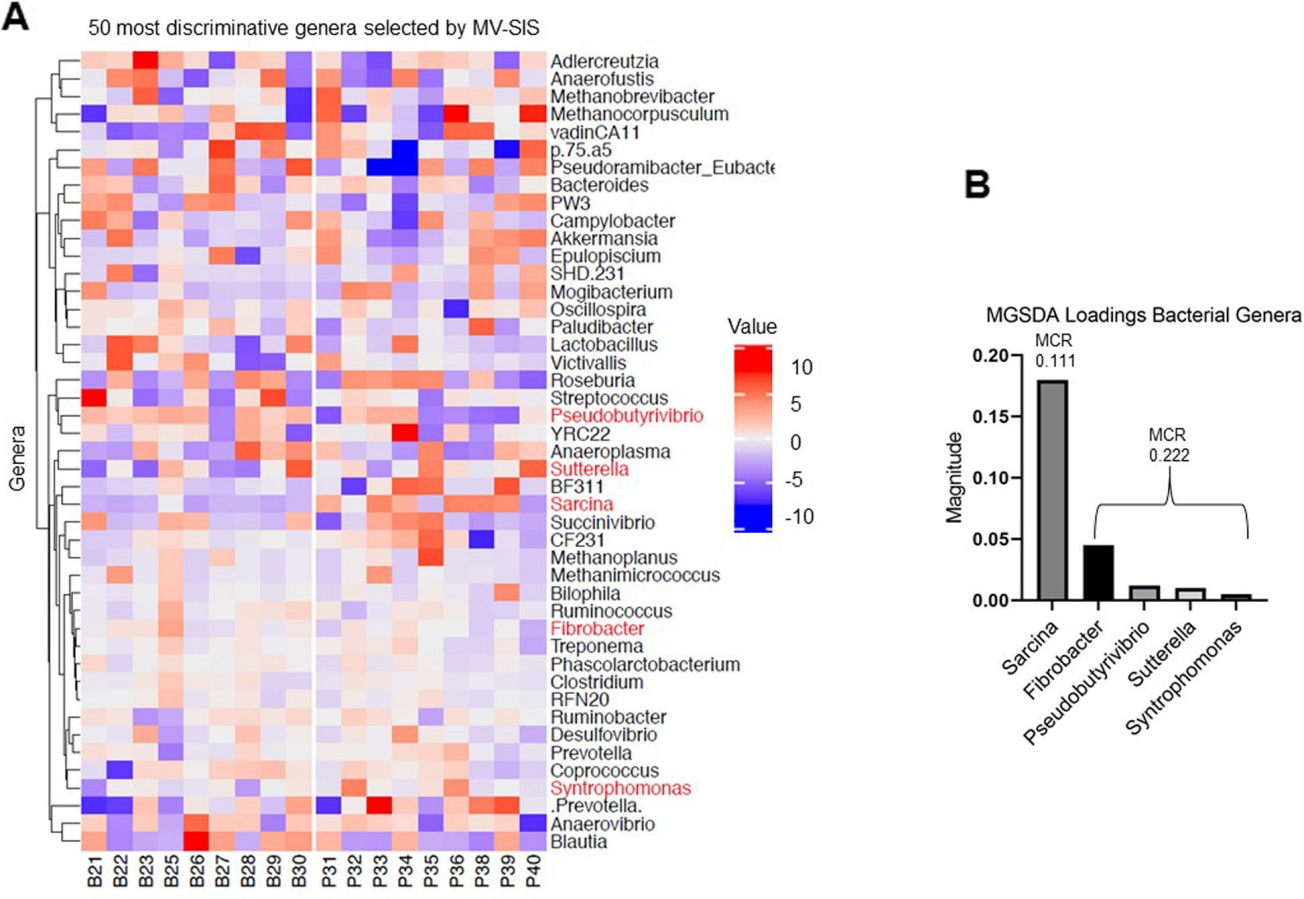
Specific fecal bacterial genera discriminate between control and NSAID-treated horses. **A** Heat map showing the distribution among the samples of the 50 most discriminative bacterial genera selected by MV-SIS. Values are scaled around zero as indicated by the key. MGSDA-selected metabolites are shown in red text. **B** Bar chart indicating the magnitude of the loadings of MGSDA-selected metabolites. The misclassification rate (MCR) is indicated by the number at the top of each bar(s)

There were 21,622 unique ASVs identified in the 16S rRNA gene sequence data. These were aggregated to the genera level for further analysis. We initially performed PCA based on relative abundance at the genus level. In order to improve visualization of the PCA, we removed genera that we present in fewer than 6 samples among the 36 samples that were available for analysis. Similar to the fecal metabolome of these horses, there was overlap of both groups at day 0 and clear visual shifts from day 0 to day 10 for both groups, although the direction of the population changes were different between the groups (Fig. 3A). Feature screening with MV-SIS was initially performed (Fig. 4A). MGSDA was applied to the features and the genus *Sarcina* was selected as the best discriminator of groups with an error rate of 0.11. Selection of the next most informative genera, *Fibrobacter*, *Pseudobutyrivibrio*, *Sutterella*, and *Syntrophomonas* increased the error rate to 0.22 (Fig. 4B). The contribution of each bacterial genus to group separation on the PCA is demonstrated in the biplot along with the percent relative abundance of these genera (Fig. 2A and B). Taken together, these findings highlight the importance of the bacterial genera selected by MGSDA.

#### Gastrointestinal transcriptome

**Fig. 5:**
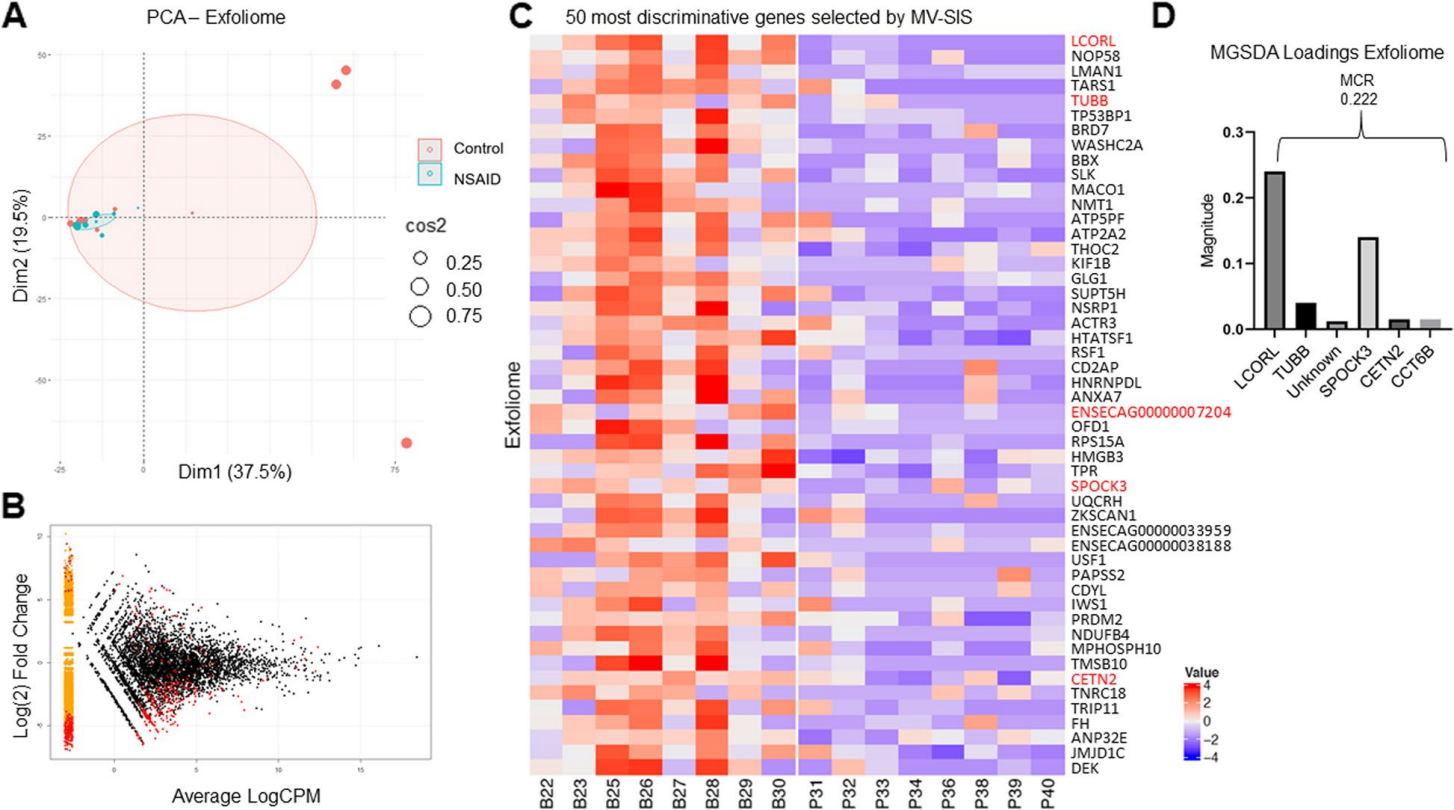
The equine exfoliome is altered by phenylbutazone administration. **A** PCA plot based on gene expression in the equine exfoliome after 9 days of phenylbutazone administration (NSAID) or placebo (control). Ellipses represent 95% CI around the group mean points. Point size indicates quality of representation (cos2) of individuals on the PCA; larger point size reflects higher quality representation. **B** Smear plot of the fold differences in exfoliome gene expression between NSAID and control horses. Red dots represent genes with greater than 2-fold difference between the groups. Yellow smear on the left of the graph represents genes with zero or very low counts in one group but not the other. **C** Heat map showing the distribution among the samples of the 50 most discriminative genes selected by MV-SIS. Values are scaled around zero as indicated by the key. MGSDA-selected metabolites are shown in red text. **B** Bar chart indicating the magnitude of the loadings of MGSDA-selected metabolites. The misclassification rate (MCR) is indicated by the number at the top of each bar(s)

The RNA isolated from 2 samples, one from each group, was of insufficient quality to proceed with sequencing and therefore, exfoliome data from 8 horses in each group was analyzed. These 16 horses mapped to 14,092 genes out of the 30,000 genes in the EquCab 3.0 genome. PCA plots of the global transcriptome revealed nearly complete overlap of both groups; however, the NSAID group was tightly clustered whereas the control group was widely dispersed (Fig. 5A). In addition, many of the most informative genes were downregulated in the NSAID group relative to the control group (Fig. 5B), although there were no differences in library size between the groups nor expression of house-keeping genes (Supp. Figure 3). We next employed MV-SIS to screen the exfoliome data (Fig. 5C). MGSDA selected 6 genes with a misclassification rate of 0.125, although LCORL was the most informative (Fig. 5D). In addition, LCORL was highly correlated (*R* > 0.95) to 10 other genes that were also selected by MV-SIS (Table 1). Therefore, these additional genes were not selected by MGSDA because they were considered redundant due to their high correlation but possess similar discrimination ability as LCORL.

**Table 1:** List of features identified as being most informative in our model. These include features (i.e., bacterial genera, metabolites, and genes) that were selected by all analytical approaches (bolded) and those that were added based on magnitude of JACA loads or high correlation to the top MGSDA selected feature

Exfoliome	
**LCORL**	**Selected by all**
**TUBB**	**Selected by all**
**TARS1**	**Selected by all**
SPOCK3	JACA rank 4
HTATSF1	JACA rank 5
LMAN1	JACA rank 6
ZNF782	JACA rank 3
GGNBP2	LCORL cor
PSMD1	LCORL cor
RPL15	LCORL cor
ERBIN	LCORL cor
TRIP11	LCORL cor
LMAN1	LCORL cor
SMURF2	LCORL cor
PRPF4B	LCORL cor
KMT2E	LCORL cor
RPL7	LCORL cor
Microbiota	
***Sarcina***	**Selected by all**
***Pseudobutyrivibrio***	**Selected by all**
*Fibrobacter*	JACA rank 3
*Syntrophomonas*	JACA rank 4
Metabolome	
**2,4,6-Trihydroxybenzoate**	**Selected by all**
**2-Hydroxynervonate**	**Selected by all**
**Methyladipate**	**Selected by all**
**Gamma-aminobutyrate**	**Selected by all**
(3′-5′)-Adenylyluridine	JACA rank 3
Kynurenine	JACA rank 2
n-Acetylhistidine	JACA rank 2
Anthranilate	JACA rank 5

### Data integration and biological interpretation

**Fig. 6:**
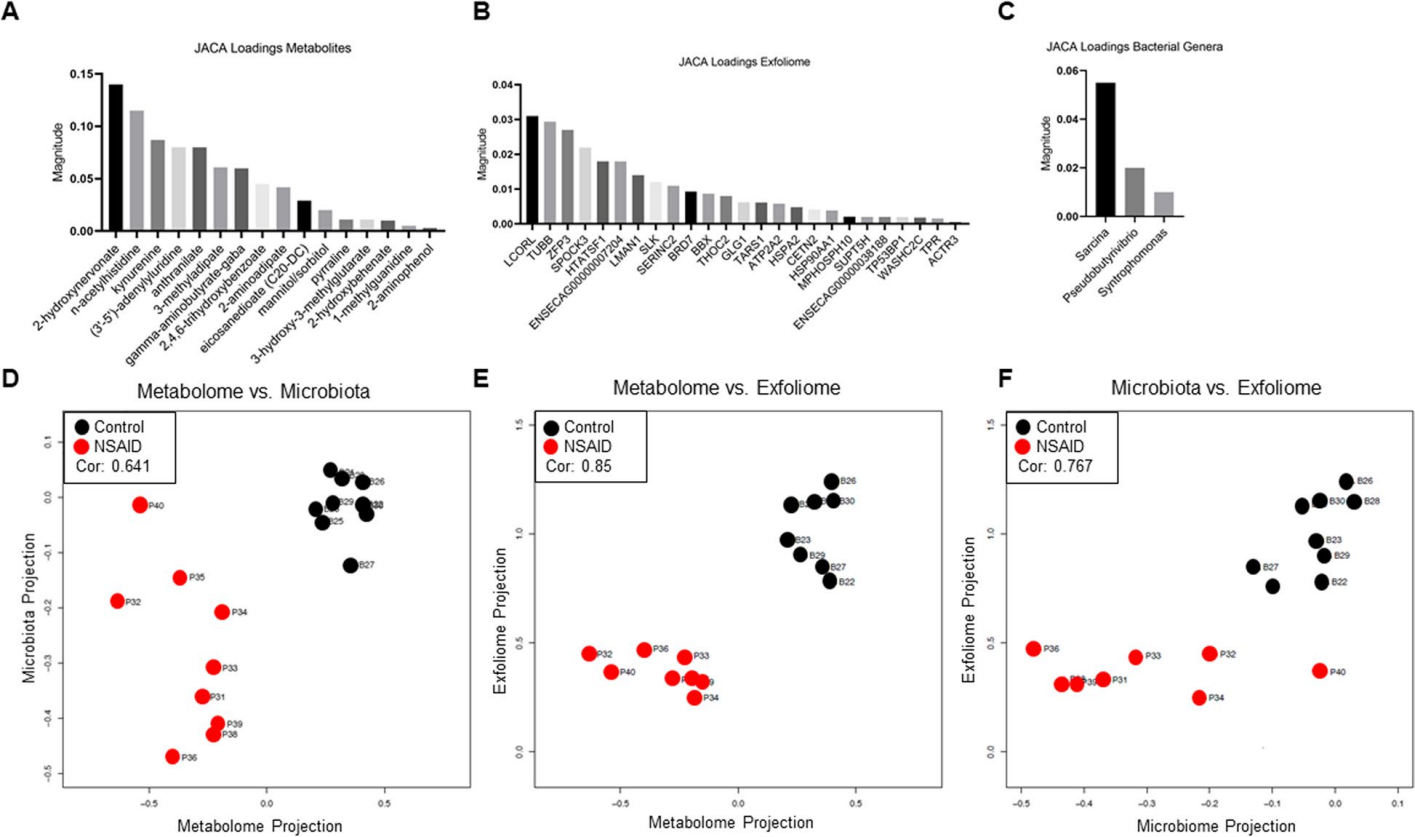
JACA identifies features that correlate with features in all three datasets and discriminate between groups. JACA-selected features and the magnitude of the loadings of each feature, which indicates both ability of that feature to discriminate between the groups and its correlation to features in the other 2 datasets, for **A** metabolome, **B** exfoliome, and **C** microbiota. Pairwise correlation of JACA-selected features and projection of samples in the direction of JACA-selected features for **D** metabolome vs. microbiota, **E** metabolome vs. exfoliome, and **F** microbiota vs. exfoliome

Ultimately intestinal biology involves a complex interaction between the microbiota and the host, combined with their respective contributions to the intestinal metabolome. To elucidate the interaction between these sets of data (i.e., microbiota, exfoliome, and metabolome), we performed JACA. JACA jointly identifies discriminative variables from the combined three data sets [34]. This analytical platform provides coherent information to the model in that signals associated with selected variables have high correlation across data sets. Ultimately, 3 bacterial genera, 16 metabolites, and 25 host genes were selected by JACA (Fig. 6A–C). Pairwise projections of the samples in the direction of the selected features revealed clear separation of the groups (Fig. 6D–F). The correlation of the exfoliome and metabolome was strong (0.85) as was the correlation between exfoliome and microbiota (0.76). The correlation between the metabolome and microbiota was moderate (0.64).

**Fig. 7:**
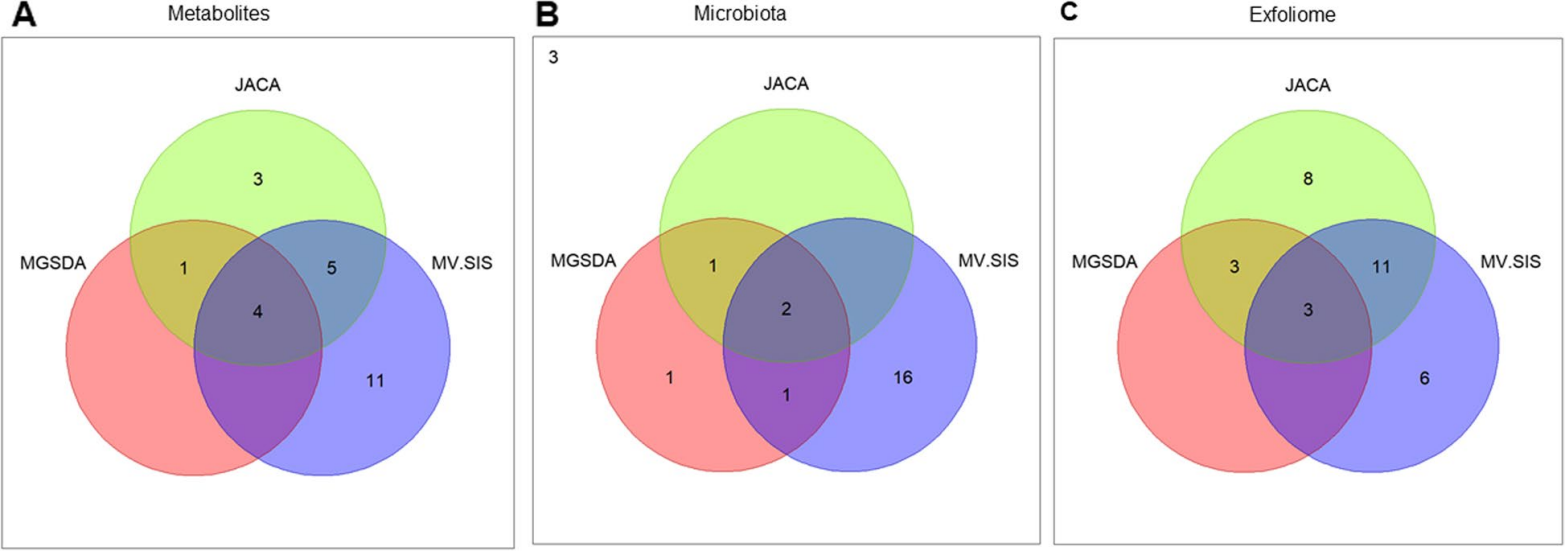
Venn diagram depicting the congruency of features selected by our analytical approaches for the **A** metabolome, **B** microbiota, and **C** exfoliome

Traditional statistical approaches that attempt to identify features that are differentially expressed or abundant provide useful information but results of these traditional analyses with ultra-high dimensional data and small sample size can generate suspicious findings due to the size and inherent noise in these types of data. Therefore, we attempted to overcome this by utilizing analytical techniques which were designed to address these specific limitations. Each analysis provided different information although there was substantial overlap of the results (Fig. 7A–C). Since discriminating features commonly selected by multiple techniques are likely to be the most robust, we built our mechanistic hypothesis around features that were commonly selected by all analytical techniques with additional features added to this model based on 2 criteria: (1) highly correlative (*R* > 0.95) to the top MGSDA/JACA and MV-SIS features and (2) the top 1/3 of JACA-selected features based on magnitude of loadings. This feature selection approach resulted in identification of 8 metabolites, 4 bacterial genera, and 17 host genes (Table 1). We then explored these features to identify patterns that might be informative regarding host-microbiota interactions in our model.

**Fig. 8:**
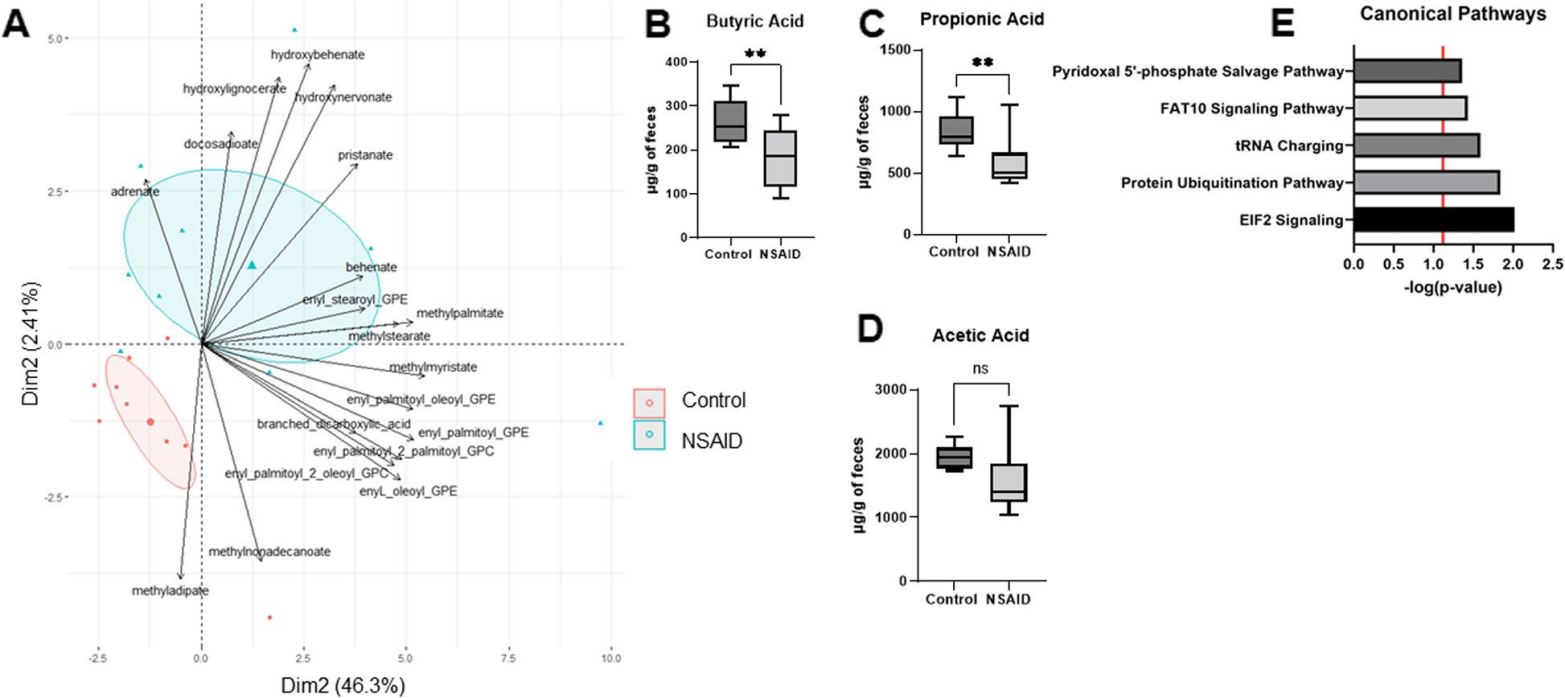
In-depth exploration of the most informative features selected by our analytical approach. **A** PCA biplot based on metabolites known to be impacted by peroxisomal dysfunction grouped by treatment (control or NSAID). Ellipses represent 95% CI around the group mean points. Point size indicates quality of representation (cos2) of individuals on the PCA; larger point size reflects higher quality representation. **B** Fecal concentration (µg/g of feces) of the SCFA butyrate was significantly lower (*P* = 0.009, independent *t*-test) in NSAID-treated horses compared with control horses. **C** Fecal concentration (µg/g of feces) of the SCFA propionate was significantly lower (*P* = 0.002, independent *t*-test) in NSAID-treated horses compared with control horses. **D** Fecal concentration (µg/g of feces) of the SCFA acetate was not significantly different between the groups

Interestingly, of the 8 metabolites selected, 2 were exclusively metabolized by peroxisomes, the VLCFA 2-hydroxynervonate and the phyanytic acid derivative 3-methyladipate. Based on these findings, we extracted the metabolites from our data that are routinely assayed for the clinical diagnosis of peroxisomal disorders in people including VLCFAs, branch chain fatty acids, plasmalogens, pristanic acid, and phytanic acid in order to examine this entire family of metabolites. PCA based on these 19 metabolites showed clear separation of the groups suggesting that this family of peroxisomal metabolites was altered by NSAID administration (Fig. 8A). An additional 2 of the 8 metabolites were tryptophan metabolites. Similar to peroxisomal metabolites, the family of tryptophan metabolites also showed clear separation of the groups (Supp Figure 4). The bacterial genera selected by our strategy were *Sarcina*, *Pseudobutyrivibrio*, *Syntrophomonas*, and *Fibrobacter*. Of these, the known function of *Pseudobutyrivibrio* production of the short chain fatty acid (SCFA) butyrate suggests that loss may have important implications for intestinal health. In order to determine if loss of this genus also resulted in decreased butyrate as expected, targeted metabolomic analysis of the primary SCFAs (i.e., propionate, butyrate, and acetate) at day 10 of both groups was performed. In concordance with loss of *Pseudobutyrivibrio*, butyrate was decreased in the feces of NSAID-treated horses (*P* = 0.009) relative to control horses as was propionate (Fig. 8B–D). Ultimately, 17 host genes met criteria for inclusion for biological interpretation. The top canonical pathways enriched by these 17 genes were EIF2 signaling and the protein ubiquitination pathway (Fig. 8E).

**Fig. 9:**
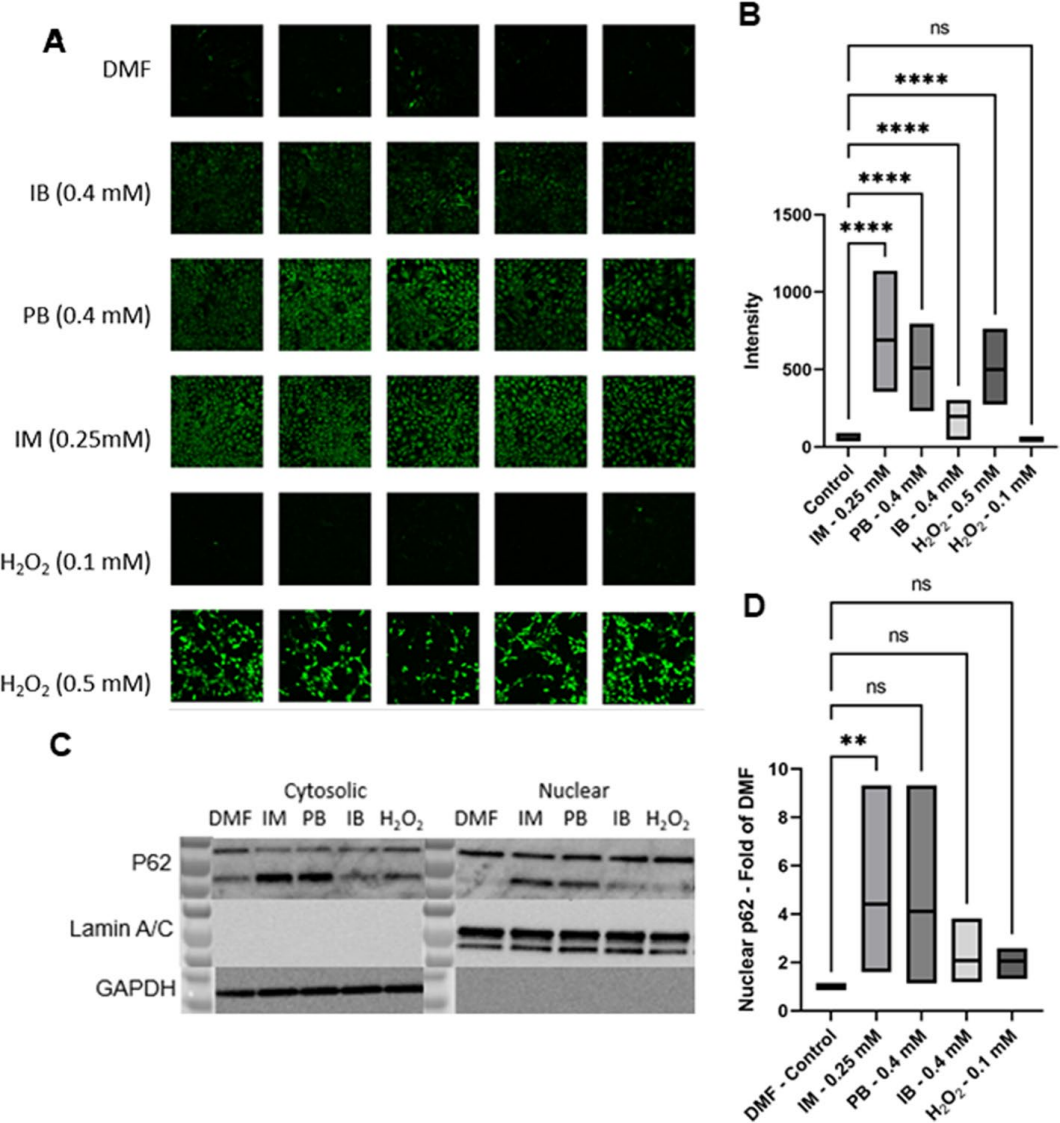
In vitro data from mouse colonocytes suggests that NSAIDs, including phenylbutazone, induce oxidative stress and a subsequent cellular response known to occur when ER stress is induced by imbalanced redox homeostasis. **A** Fluorescent microscopic images of YAMC cells exposed to NSAIDs and H_2_O_2_, at the noted concentration, for 24 h prior to exposure to the ROS indicator CM-H2DCFDA. Ten images were taken from each treatment condition. **B** Average intensity of fluorescence for each treatment condition was significantly different that control cells (ANOVA) except the lowest concentration of H_2_O_2_. Graph represents data from 3 independent experiments. **C** Western blot of P62 protein from both the cytosolic (left blot) and nuclear (right blot) protein fractions of YAMC cells exposed to the NSAIDs at the indicated concentration for 24 h. Loading controls were the nuclear protein lamin A/C and the cytosolic protein GAPDH. **D** Graph of nuclear p62 represented as fold of DMF control from 3 independent experiments. DMF: dimethylformamide (0.04%), IB: ibuprofen (0.4 mM), PB: phenylbutazone (0.4 mM), IM: indomethacin (0.25 mM), H_2_O_2_ (0.5 or 0.1 mM as indicated)

**Fig. 10:**
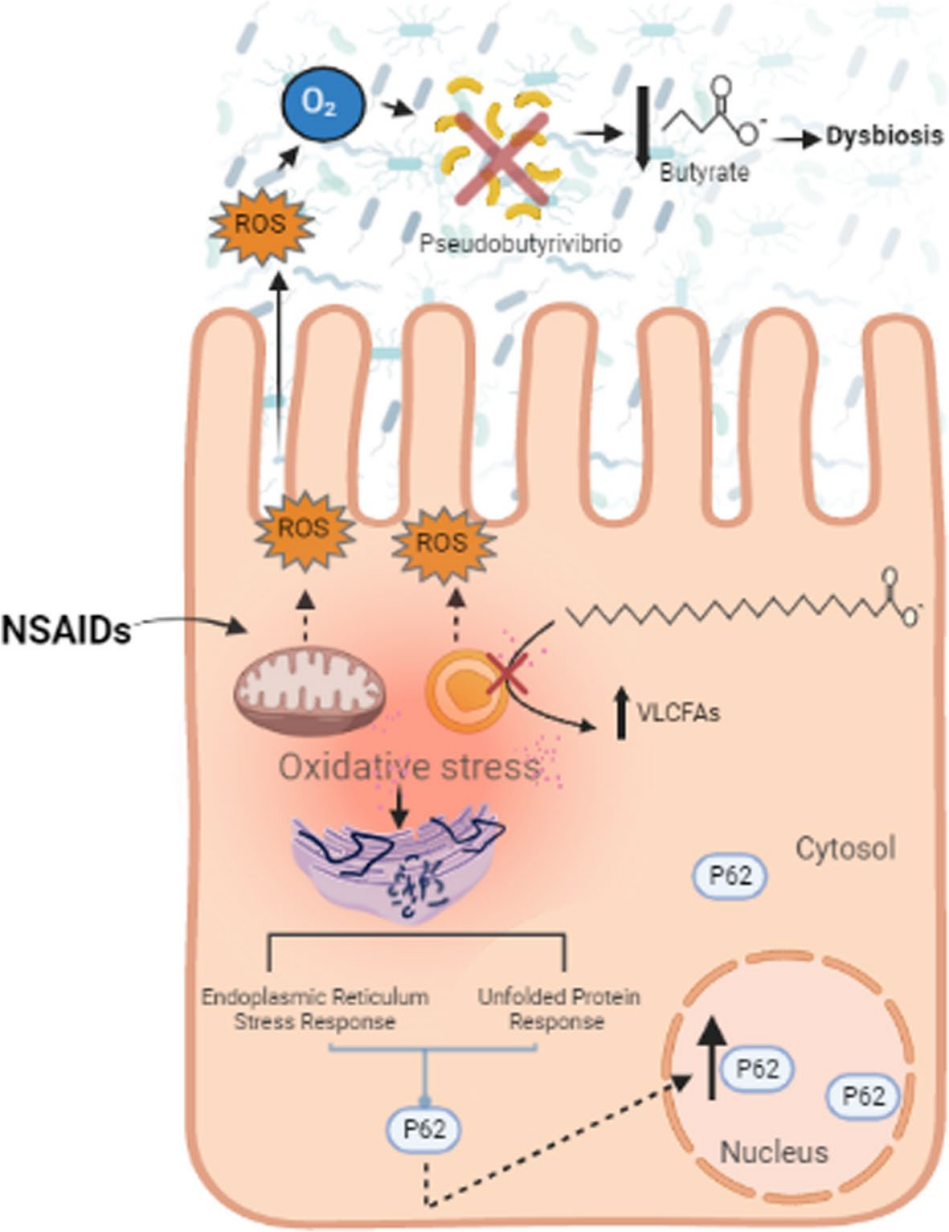
Putative mechanism describing how phenylbutazone induces injury in the GI tract. We propose that phenylbutazone induces oxidative injury to colonocytes which subsequently alters several cell signaling responses including ER stress and activation of the proteasome ubiquitin system. Further, this combination of host changes results in concommitant alteration of the microbiome, potentially due to lumenal redox imbalances. Obligate anaerobic bacteria, and their metabolites (*e.g.,* butyrate), are then depleted due to their sensitivity to lumenal oxygen content. Loss of the critical SCFA butyrate then exacerbates cellular injury

*Pseudobutyrivibrio* is an obligate anaerobic bacteria [39] and therefore, is sensitive to changes in intestinal oxygenation. Oxidative injury within the intestinal mucosa is one mechanism that has been shown to alter oxygenation status and loss of commensal anaerobic bacteria. NSAIDs are known to induce oxidative injury; however, the ability of phenylbutazone to induce oxidative injury relative to other NSAIDs has not been examined. Thus, we compared NSAID-induced ROS accumulation in young adult mice colonocytes (YAMC) cells treated with phenylbutazone and 3 other commonly used NSAIDs (Fig. 9A and B). The oxidative capability of phenylbutazone was similar to the other classes of NSAIDs examined suggesting that phenylbutazone induces similar oxidative stress as other NSAIDs. Cellular oxidative stress induces many cellular responses including endoplasmic reticulum (ER) stress [40]. Typically, the cellular response to ER stress is to increase protein degradation through induction of the ubiquitin proteome system and to decrease protein translation through the eukaryotic translation initiation factor 2 (eIF2) pathway, the top two canonical pathways enriched in this model. One cellular indication of ER stress is nuclear accumulation of p62 that occurs to increase the efficiency of the ubiquitin proteome system [41]. Interestingly, we observed nuclear accumulation of p62 in mouse colonocytes treated with various NSAIDs including phenylbutazone (Fig. 9C). Taken together, these data suggest that, in vitro, phenylbutazone induces oxidative injury to colonocytes and that the resulting cellular response may indicate ER stress and activation of the proteasome ubiquitin system. Based on these data, we have generated a putative mechanism describing the effects on NSAIDs with respect to GI injury (Fig. 10).

## Discussion

Non-invasively acquired data regarding the cellular and molecular function of the GI tract has broad implications in the field of gastroenterology for all animal species. Fecal microbiota data have been examined for decades in the context of GI diseases in both people and animals. While descriptive microbiota data can provide useful information, linking microbiota changes to intestinal function increases the value of such data. The major limitations of linking microbiota data and GI functional information are the difficulties of acquisition of cellular and molecular data regarding the GI tract and the challenges of computational analysis of these large data sets. We combined novel techniques (i.e., exfoliomics) and robust computational approaches to integrate host microbiota data in an equine model of NSAID-induced GI injury. Our findings recapitulating known mechanisms of NSAID-induced GI injury provide proof-of-principle for the validity of our non-invasive approach to investigate GI diseases in both humans and animals.

Of the many animal models of GI inflammation, chemically induced models are among the most common. Each of these models has advantages and disadvantages and these models have been extensively reviewed elsewhere [42, 43]. NSAIDs have been used as a chemically induced model of GI injury [44, 45]. NSAID-induced GI injury is an attractive model because it is a clinically relevant condition [46, 47] and shares many pathological features with other IBDs [48]. As with IBD, microbiomic changes are a key feature of NSAID enteropathy, thus enabling use of the NSAID model for examination of host-microbiota interactions [49]. Although much of this work has been conducted in mice and rats, both clinical cases and the equine model of NSAID-induced GI injury have GI lesions similar to those in people and mice [17, 18]. While the equine model has limitations, large animal models offer important benefits [50]. We used the model of NSAID-induced GI injury [17, 18] in horses and our experience with equine exfoliomics [9] and microbiomics [19] to integrate host and microbiota data to gain insights into the pathogenesis of GI injury.

The most commonly accepted paradigm for lower GI injury begins with NSAID accumulation within intestinal epithelial cells (IEC). NSAIDs are weak organic acids [51] that can easily traverse the plasma membrane of IECs. Intracellularly, NSAIDs induce mitochondrial injury and subsequently oxidative stress [52]. This has been well-documented for the cyclooxygenase (COX) non-selective NSAID indomethacin, the most common NSAID utilized in animal models of GI injury. The ability of phenylbutazone to induce ROS in IECs has not been well-studied; however, our results demonstrate that phenylbutazone induces ROS accumulation. This is consistent with other studies where phenylbutazone induced ROS in other tissues [53, 54].

Oxidative stress induces a myriad of cellular responses that are highly cell- and context-dependent. The metabolite identified by all of the analytical approaches was the VLCFA 2-hydroxynervonate which was increased in NSAID-treated horses relative to controls. Accumulation of VLCFAs is a hallmark of peroxisomal dysfunction. While inherited peroxisomal disorders have been described in humans, acquired disorders are more common. One of the few defined causes of acquired peroxisome disorders is oxidative stress which has been demonstrated in diabetes [55] and aging [56]. In our model, oxidative stress appears to be the most likely cause given our findings and the known effects of other NSAIDs on redox homeostasis [57]. Peroxisomes are critically involved in redox homeostasis, generate large amounts of cellular hydrogen peroxide [58], and can be overwhelmed during oxidative stress resulting in dysfunction [59, 60]. While we had no means of examining peroxisome function in this study, we were able to examine other metabolites associated with peroxisomal biogenesis disorders. These included plasmalogens, synthesized in peroxisomes and terminated in the ER [61], and other VLCFAs [62]. A notable difference in this class of metabolites was observed between the groups. Others have demonstrated similar findings associated with peroxisomal dysfunction in numerous diseases including Alzheimer’s disease [63], Zellweger syndrome [64], and various types of cancers [65].

As noted, oxidative stress induces a wide array of cellular responses. The majority of ROS are generated by the mitochondria (70%), with peroxisomes providing much of the remaining 30% [66]. There is well-recognized cross-talk between peroxisomes and mitochondria in terms of fatty acid metabolism for energy and redox homeostasis among others [67]. Peroxisomes and mitochondria are in close proximity to the ER and contact the ER through mitochondrial-associated membranes. ROS generated by these organelles diffuse to the ER and induce a stress response [55], including the accumulation of misfolded proteins within the cell. ER stress initiates a highly conserved cell-signaling pathway referred to as the unfolded protein response pathway (UPR). The UPR attempts to restore protein folding capacity of the cell via a series of cell signaling transduction events with 4 goals that vary based on the severity of oxidative stress and other factors. These goals are (1) a global decrease in protein synthesis, (2) increased ER folding capacity, (3) increased degradation of misfolded proteins, or (4) cell death, if uncorrected. Clearly, these diverse and broad cellular responses involve a complex interaction of many genes, proteins, and transcription factors.

The host genes selected in our model can be grouped based on cellular function into 4 distinct, but overlapping outcomes: (1) ER to Golgi trafficking (TRIP11, LMAN1); (2) protein degradation through autophagy and ubiquitination (ERBIN, PSMD1); (3) cell cycle regulation (KMT2E); (4) transcription regulation (ZNF782, PRP4FB); and (5) EIF2 signaling (RPL15, RPL7). Each of these functions is well described events associated with the UPR and suggests that NSAIDs induce ER stress, possibly through imbalance of redox homeostasis, and induction of UPR with associated downstream functions. While these findings are known to occur with oxidative stress in general, NSAIDs have been shown to induce ER stress [68] and associated downstream effects including UPR [69]. The mechanisms by which NSAIDs induce ER stress are unclear but oxidative stress with mitochondrial dysfunction and effects of NSAIDs on cell membranes have been implicated [70].

One outcome of activation of UPR is enhanced protein degradation though proteosomal degradation and/or autophagy. Many classes of NSAIDs have been shown to impact macroautophagy although whether NSAIDs inhibit or induce macroautophagy is unclear. Interestingly, ERBIN expression is decreased in people with IBD, and ERBIN inhibits autophagy and subsequent autophagic cell death in murine models of DSS-induced colitis [71]. In our study, the expression of ERBIN was downregulated in NSAID-treated horses, consistent with other GI inflammatory diseases. p62 is a protein that is critically involved in the intersection of autophagy and proteosomal degradation. This protein binds to ubiquitinated targets resulting in autophagic degradation. p62 is also important for pexophagy, the process by which cells remove dysfunctional peroxisomes. Notably, our data demonstrate that phenylbutazone increases the amount of p62 and results in nuclear translocation of p62. This is noteworthy, as p62 is specifically required for pexophagy during oxidative stress conditions [72, 73]. Taken together, our metabolomic and exfoliomic data highlight the role of redox homeostasis and subsequent ER stress in phenylbutazone-induced intestinal injury in horses.

While redox homeostasis is important in all cells, the impact of imbalances is pronounced in the GI tract and has been associated with many GI diseases. One reason for this pronounced effect is that, unlike other tissues in the body, the GI mucosa is hypoxic under homeostatic conditions. There are structural and physiological reasons for this, including the maintenance of an anaerobic environment in the lumen of the GI tract. The GI microbiota is a large and diverse system with the vast majority of the bacteria known to be facultative or obligate anaerobes [74]. Redox imbalances can result in increased oxygen levels within both the GI mucosa and lumen allowing for increased growth of aerobic bacteria and loss of obligate anaerobes [75]. In our study, one of the bacterial genera identified by multiple computational approaches was *Pseudobutyrivibrio*, which was decreased in horses after NSAID treatment. The reasons for this decreased abundance are unclear and, while redox imbalance may have played a role, that cannot be the only explanation as other obligate anaerobes increased in relative abundance in response to NSAIDs. However, loss of *Pseudobutyrivibrio* may have contributed to disease severity due to loss of SCFA butyrate, which is produced in large quantities by *Pseudobutyrivibrio* [76]. We confirmed that loss of this genus was associated with loss of the SCFA butyrate. Butyrate is an important bacterial metabolite with a broad array of impacts on GI mucosal homeostasis including potent antioxidant activity [77, 78]. Butyrate also directly impacts peroxisome proliferation and function in IECs [79]. Therefore, phenylbutazone-induced oxidative injury combined with decreases in one of the major antioxidant metabolites (i.e., butyrate) may have exacerbated redox imbalances in a vicious cycle and further induced peroxisome injury and ER stress, ultimately leading to cell death and intestinal injury.

*Sarcina* was another genus of bacteria that was identified by all computational methods and was the best discriminator of the groups. This genus is found in the feces of normal healthy humans [80] and other animals including horses [81]. However, it has also been implicated in severe gastritis and gastric rupture in humans [82] and animals [83]. Whether it causes these pathologies or is simply an opportunistic pathogen remains unclear. NSAID-induced gastric ulcers are common [17, 84] and the horses in this study developed gastric ulcers as expected. Because *Sarcina* is a component of the normal flora of the equine stomach [85], it is possible that the increased abundance of *Sarcina* in NSAID-treated horses may be due to colonization of NSAID-induced gastric ulcers.

The role of the microbiota in GI diseases is well-recognized and many descriptive studies link microbiota changes with various GI diseases. Elucidating host-microbiota interactions from a cellular and molecular perspective, however, is challenging. We attempted to address this challenge by combining host, fecal metabolomic, and fecal microbiota data. The power of our study lies in the robust computational analysis used for integrating host and microbiota data. Identifying the features that are biologically important of high-dimensional data, especially with a small sample size, is challenging. Traditional approaches that attempt classification using all features in large datasets can be little more than a guess due to the noise inherently present in high-dimensional data [86]. Our approach employed multiple analytical methods followed by focusing on features that were commonly identified among these techniques. Ultimately, this methodology identified a sparse set of features from each of our datasets (i.e., metabolites, bacterial genera, and host genes) that were available for biological interpretation. Biological interpretation of our findings recapitulates many of the known or suspected initiators of NSAID-induced intestinal injury which leads credence to this analytical approach. Importantly, the mild degree of intestinal injury induced by our model allowed us to recognize of initiating events of NSAID enteropathy. Many studies utilize models of NSAID enteropathy that induce severe injury which has provided a wealth of information about mucosal injury and subsequent inflammatory cascade. In these studies, however, the severe inflammatory reaction can mask the initiating events. Identification of pathways involved in initiating events can lead to avenues for therapeutic intervention designed to prevent NSAID enteropathy and, perhaps, other GI diseases characterized by imbalances in intestinal redox homeostasis.

The majority of changes observed in all 3 datasets can be traced to oxidative stress within the GI tract and associated metabolite, microbiota, and gene expression changes. However, other changes in these datasets merit discussion. Changes were observed in 2 of the 8 selected tryptophan-derived metabolites (viz., kynurenine and anthranilate). Tryptophan and both its mammalian- and microbiota-derived metabolites have been extensively studied in the context of immunoregulation and various types of IBDs. While some results are conflicting, multiple authors have demonstrated increased levels of fecal tryptophan and increased tryptophan metabolites in cases of active IBD [87]. This likely reflects some combination of increased tryptophan metabolism, decreased absorption, or increased loss from injured GI tissues [87, 88]. We and others have previously examined the effects of tryptophan metabolites in a murine model of NSAID-induced intestinal injury and demonstrated interactions between NSAID-induced injury and tryptophan metabolites [16]. The congruency of these findings further supports the importance of this family of metabolites in GI inflammation.

There were several limitations to our study that should be recognized. First, the sample size for this study was small (*n* = 9 for fecal-based assays) and in some readouts (e.g., exfoliome) the sample size was further decreased by logistical issues such as poor RNA quality. Due to our small sample size, we attempted to remove as many other variables between the groups as possible. For example, 75 days of acclimation, same diet, and side-by-side housing to name a few. Despite these steps, there were still differences between the groups at the start of this study as evidenced by differences at day 0 for both the metabolome and microbiota. While we attempted to house horses as similar as possible, it is possible that housing differences, even as minimal as adjacent pens, resulted in different baseline findings. The combination of small sample size and starting with a different baseline metabolome and microbiota may have limited our ability to detect other important biological signatures. Another point that is related to group assignment is the fact that there were also changes in the control group over the study period. This suggests that NSAIDs were not the only cause some of the changes we observed. The reasons for the changes in the control group are unclear but may be related withholding feed twice within the 10 day treatment period as diet changes have been shown to alter the fecal microbiota [89].

Other limitations were related to our sample acquisition and analyses. We used 16S rRNA gene sequencing of fecal samples for microbiota analysis. There are many well-described limitations to this approach related to taxonomic resolution, lack of functional information, and inability to assign taxonomy to a large proportion of ASVs [90]. This is further compounded by the fact that we used fecal samples only. The ability of fecal samples to represent the microbiota of the proximal GI tract is questionable at best. Relatedly, we propose a mechanistic hypothesis for NSAID-induced intestinal injury in horses, but no additional readouts were performed to confirm our hypothesis. The primary reason for that is that samples from horses were acquired non-invasively and therefore no tissues were available for confirmation nor were microbiota samples representative of the proximal GI tract available. Further, host transcriptomic data was based on analysis of the equine exfoliome. We and others have used this approach in rodents [5], pigs [6], people [7], human neonates [8], and horses [9], and demonstrated that exfoliomic data mimic those in tissues but further validation of the exfoliomic methods in horses is warranted. Finally, the portion of our mechanistic hypothesis generated by evaluation of the exfoliome is based on the function of a small number of genes that are not master regulators of the pathways we identified. For example, the well-described initiators of UPR are inositol-requiring enzyme 1α (IRE1α), pancreatic endoplasmic reticulum kinase (PERK), and activating transcription factor 6 (ATF6) [91]; thus, it is logical that our analysis should have identified an association between these master regulators and NSAID administration. None of these canonical drivers of UPR, however, was selected by our methodology. This might be attributable to the fact that interrogated changes in mRNA expression; RNA-Seq does not identify initiation events such as nuclear translocation or phosphorylation. This might also be attributed to some of the other limitations mentioned above related to the concern that the samples we collected (i.e., feces) were not highly representative of the host and microbiota responses that occurred in the proximal GI tract resulting in missing some key cellular and molecular signatures.

## Conclusions

In summary, our work demonstrates the power of non-invasive, multiomic approaches and robust computational analyses to integrate omic methods to interrogate host-microbiota interactions. Our findings recapitulate some of the known biology and pathophysiology of NSAID-induced intestinal injury, thereby adding confidence in the validity of our approach. By leveraging a mild model of injury, we have uncovered some of the initiating events of NSAID-induced intestinal injury which are often masked in more severe models. We propose a mechanistic hypothesis based on these findings. Importantly, our findings identify putative targets for therapeutics or preventatives in the treatment of NSAID enteropathy and other inflammatory GI diseases. Additional studies are needed to confirm our findings in horses and other animal species.

### Supplementary Information

**Additional file 1: Supplementary Figs. 1, 2, 3, and 4.**

**Additional file 2: Supplementary Table 1.**

## Data Availability

The datasets generated and/or analyzed during the current study are available in the NCBI Sequence Read Archive (SRA) within Bioproject # PRJNA909273 (https://dataview.ncbi.nlm.nih.gov/object/PRJNA909273). Other data are included as additional information with this submission.
