# Supplementary material for: Integrated analysis of gut metabolome, microbiome, and exfoliome data in an equine model of intestinal injury
Source: Microbiome. 2024 Apr 15;12:74. doi: 10.1186/s40168-024-01785-1 (PMC11017594; doi:10.1186/s40168-024-01785-1)
Supplement: Supplementary file 2 — Additional file 1: Supplementary Figs. 1, 2, 3, and 4. [file 40168_2024_1785_MOESM1_ESM.pdf]

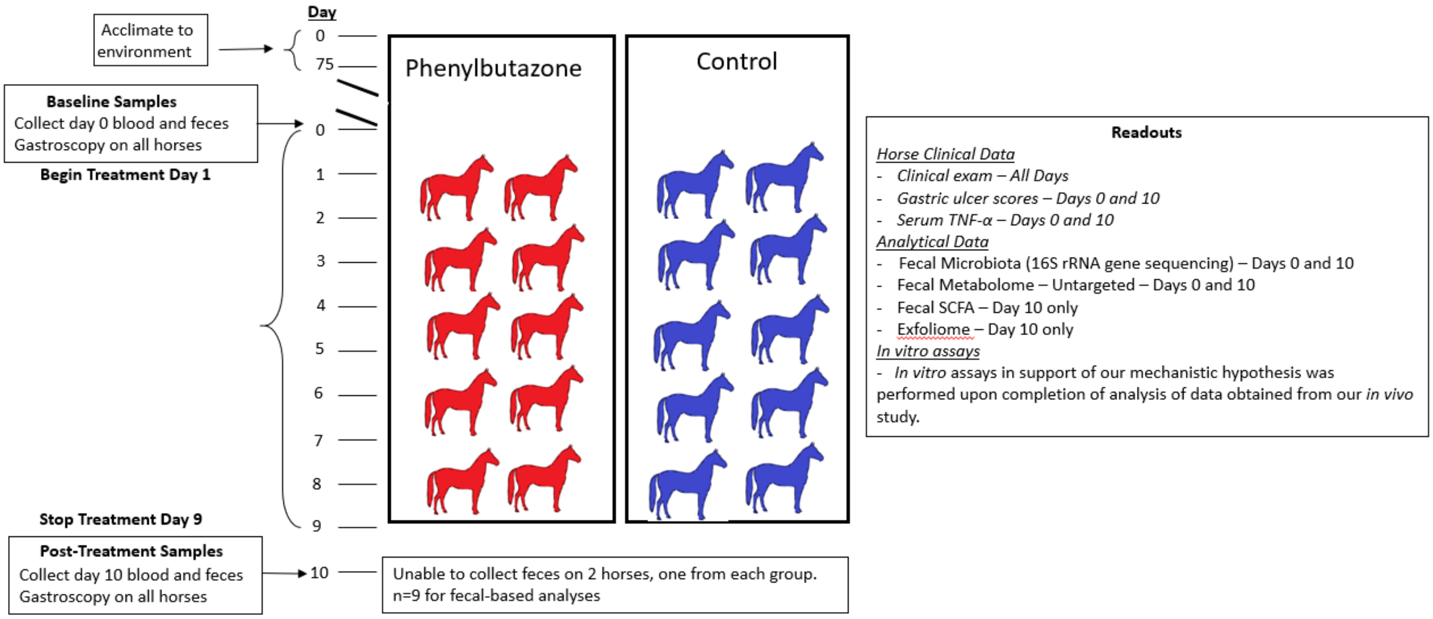

Supp Figure 1:Schematic of study design

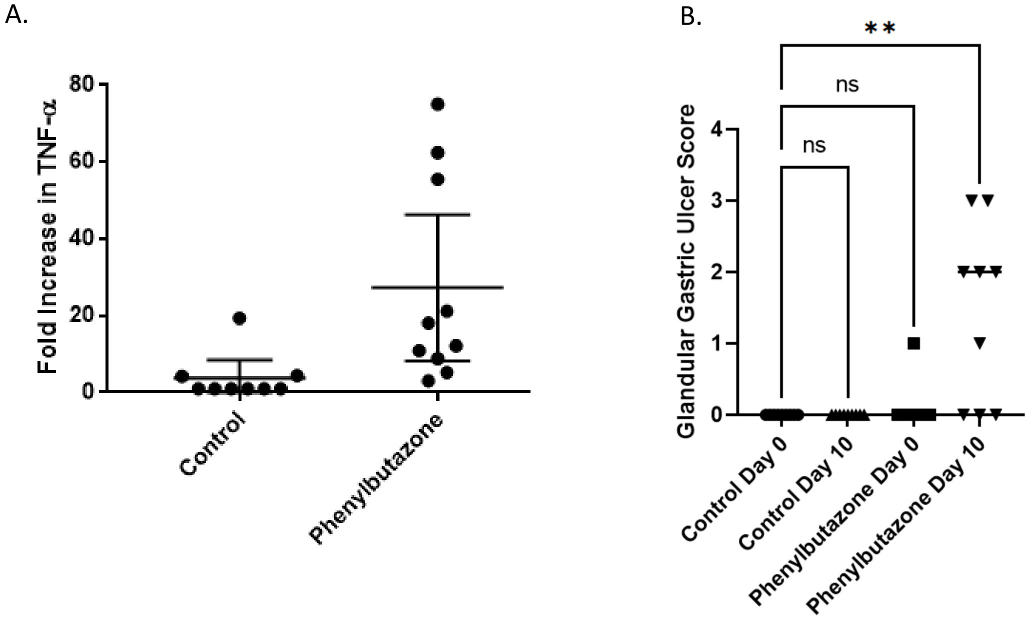

Supp Figure 2: A) Scatter plot demonstrating significant ($P < 0.05$) increase in serum TNF-α between phenylbutazone-treated horses (4.4 mg/kg orally once daily) and control (untreated) horses. Fold increase is between days 0 and 10 B) Glandular gastric ulcer scores before (day 0) and after (day 10) 9 days of phenylbutazone treatment. Horizontal bar represents median and error bars represent SD.

A.

B.

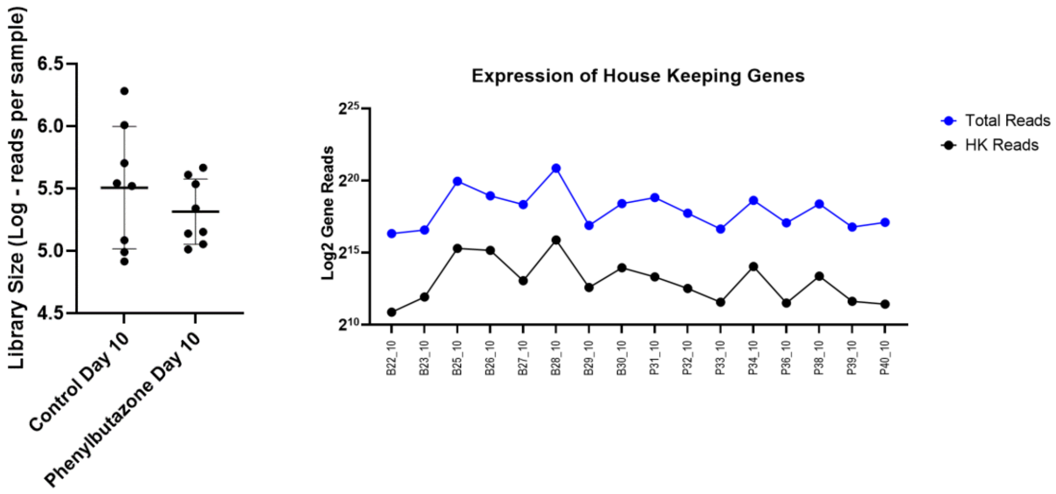

Supp Figure 3: A) Scatter plot demonstrating log transformed library size (reads per sample) between the two treatment groups. There was no difference ($P > 0.05$) between phenylbutazone-treated horses and control (untreated) horses. Horizontal bar represents median and error bars represent SD. B) Log transformed total reads per sample and house-keeping (HK) gene reads per sample. Samples are identified on the X-axis. Blue line represents HK genes, black line represents total gene reads.

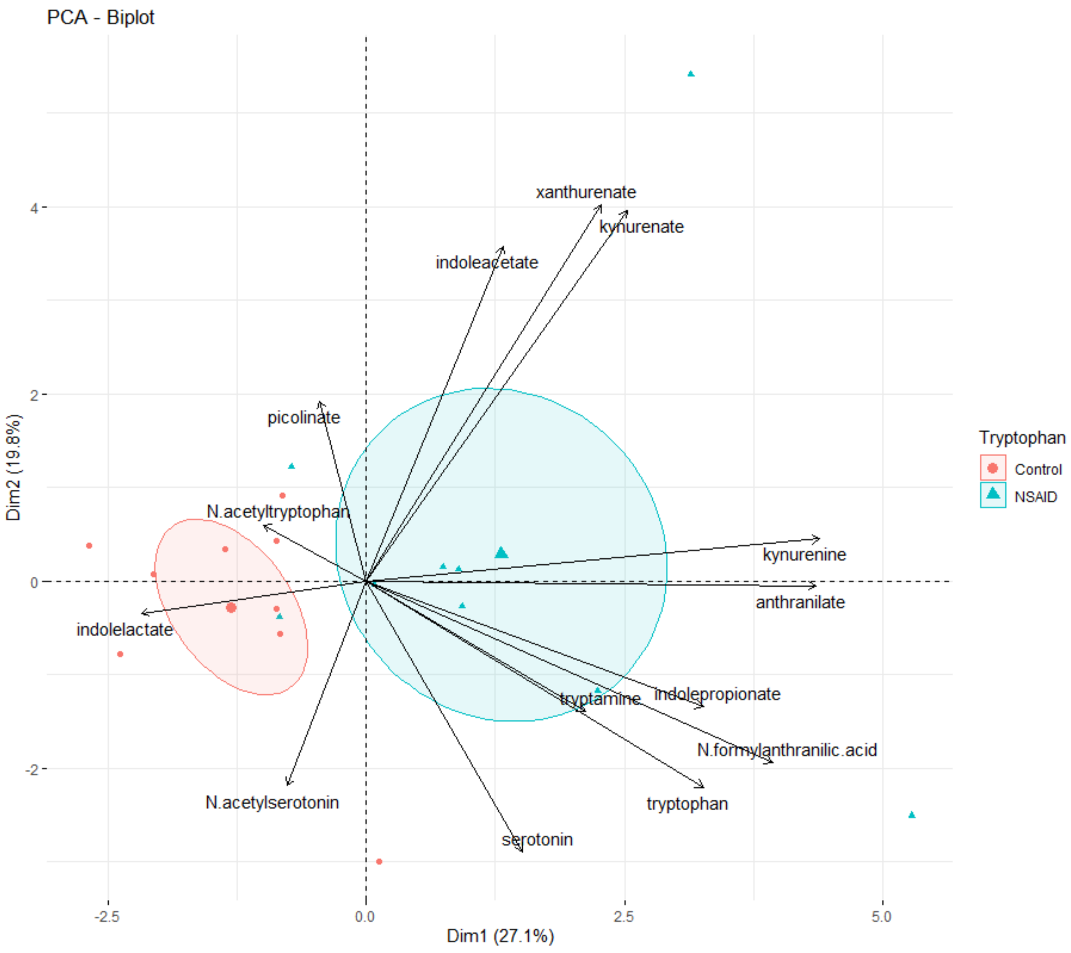

Supp Figure 4: PCA biplot based on tryptophan metabolites grouped by treatment (control or NSAID). Ellipses represent 95% CI around the group mean points. Point size indicates quality of representation (cos2) of individuals on the PCA, larger point size reflects higher quality representation.
